# Prevalence of Smartphone Addiction and Its Association with Sociodemographic, Physical and Mental Well-Being: A Cross-Sectional Study among the Young Adults of Bangladesh

**DOI:** 10.3390/ijerph192416583

**Published:** 2022-12-09

**Authors:** Zubair Ahmed Ratan, Anne-Maree Parrish, Mohammad Saud Alotaibi, Hassan Hosseinzadeh

**Affiliations:** 1 School of Health and Society, Faculty of the Arts, Social Sciences and Humanities, University of Wollongong, Northfields Ave., Wollongong, NSW 2522, Australia; 2 Department of Biomedical Engineering, Khulna University of Engineering and Technology, Khulna 9203, Bangladesh; 3 Department of Social Work, College of Social Sciences, Umm Al-Qura University, Mecca 24382, Saudi Arabia

**Keywords:** smartphone addiction, physical and mental health, prevalence, Bangladesh

## Abstract

Smartphones have made our lives easier and have become indispensable for everyday life; however, their uncontrolled and excessive use can trigger “smartphone addiction” (SA). SA is a rising public health issue, particularly among young people around the world. There is a dearth of empirical research about SA and its impacts on young adults, particularly in developing countries such as Bangladesh. This cross-sectional study is an attempt to fill this gap. The data were collected from 440 eligible young adults in Bangladesh using an online survey between July 2021 and February 2022. Study results revealed that 61.4% of the young adults were addicted to their smartphone. Logistic regression analysis showed that being male, aged ≤25, unemployed and living with a large family size (≥8) were the significant sociodemographic predictors of SA. Smartphone-addicted participants were more likely to be less physically active, suffer from insomnia, be overweight or obese and use their phones while driving, walking and eating. In addition, the SA group were more likely to have physical and mental well-being problems. This study brings to light significant implications for policy makers and indicates a need for an SA community awareness programme which aims to reduce SA at the societal level.

## 1. Introduction

Smartphones offer entertainment and socialization opportunities such as playing online games, surfing the web and browsing social media [1], as well as make life easier with their convenient software applications [2]. According to Statista in 2022, about 83.72% of the world’s population, over 6 billion people, use a smartphone [3], with young people being the most common users [4]. Despite its advantages, the smartphone brings some new challenges; people look at their smartphones while walking or driving [5], and there are reports of “nomophobia”, which was explained by Bhattacharya, Bashar [6] as “a psychological condition when people have a fear of being detached from mobile phone connectivity”.

A recent systematic review indicates that “overuse”, “problematic use” and “excessive use” have been used alternatively to narrate SA [7]. According to Ching, Yee [8], SA is “mainly characterised by excessive or poorly controlled preoccupations, usage or behaviour regarding smartphone use; to the extent that individuals neglect other areas of life”. According to Alhazmi, Alzahrani [1], “the excessive use of smartphones to a level where it interferes with the daily lives of users is considered to be smartphone addiction”. Interestingly, until now, the Diagnostic and Statistical Manual of Mental Disorders (DSM-5) has not recognized SA; however, it has recognized the “diagnostic criteria for Internet gaming addiction with the need for further research” [9]. We think SA is another form of digital addiction since, in line with Professor Griffiths [10], salience, mood modification, tolerance, withdrawal, conflicts and relapse are key components of SA.

Previous studies revealed that SA was associated with sociodemographic factors and found that young people were at higher risk of SA [11,12,13,14]. For an example, a recent study conducted in Saudi Arabia reported that youth was a strong predictor of SA [12]. Several studies also suggested that that SA was associated with different physical problems like insomnia [15], musculoskeletal problems [16] and low levels of physical activity [17]. A study conducted among Korean adults found that cervical repositioning errors were associated with SA [18]. Another recent study also revealed that SA can lead to chronic neck pain [19]. In addition, research has shown that smartphone-addicted drivers use their devices hazardously while driving [20]. Existing evidence also suggests that SA can lead to anxiety, stress and negative emotions [21,22].

Once considered a luxury, smartphones today are an essential device in every household in all countries of the world, and Bangladesh is no exception. Bangladesh has seen a high smartphone penetration rate and has become the fifth-largest mobile market in the Asia Pacific region. According to the Bangladesh Telecommunication Regulatory Commission (BTRC), there are more than 176 million people using smartphones in Bangladesh [23]. One recent study in Bangladesh reported that young people were more vulnerable to SA [24]. However, there are limited studies about SA among young adults in Bangladesh. To address this gap, this current study attempts to investigate (1) the prevalence of SA, (2) the association of sociodemographic factors with smartphone addiction and (3) the effect of SA on the physical and mental health of Bangladeshi young adults.

## 2. Methods

### 2.1. Study Design and Data Collection

This cross-sectional study was performed with young adults aged 18 to 32 years in Bangladesh from July 2021 to February 2022. Data were collected using an online survey administered via Qualtrics (Provo, UT, USA). The participants were recruited after the study was advertised via flyers, social media platforms and emails. A “Participant Information Sheet (PIS)” and “Participant Consent Form (PCF)” were included in the online survey. The online survey link was attached to the study advertisement. The survey took 15–25 min to complete. This current study was approved by the University of Wollongong Human Research Ethics Committee (HREC) in July 2021 (Reference number 2021/059).

### 2.2. Study Measures

Sociodemographic measures included age, gender, occupation status, family size, marital status, education level and family income.

Smartphone Addiction (SA) was measured using the “Smartphone addiction (SAS-SV) scale” [25]. This is an internationally validated scale [7] with 10 questions, each with a Likert scale from 1 to 6, indicating “strongly disagree” (1) to “strongly agree” (6). The overall SAS-SV score ranges from 10 to 60, and the cut-off value for addiction is different for male and female participants (31 for males and 33 for females). In this current study, the Cronbach’s α of the SAS-SV was 0.84.

Anxiety and depression were measured by the four-item “Patient Health Questionnaire (PHQ-4)”, which is composed of depression (PHQ-2) and generalized anxiety disorders (GAD) screener (GAD-2) items [26]. This four-item scale has two questions about anxiety, including “Feeling nervous, anxious or on edge?” and “Not being able to stop or control worrying?” It also includes two questions about depression, including “Feeling down, depressed or hopeless?” and “Little interest or pleasure in doing things?” The questions are scored from 0 (not at all) to 4 (nearly every day) [26]. Respondents with GAD-2 and PHQ-2 scores of ≥3 have been advised to seek further screening for anxiety and depression, respectively [27]. The Cronbach’s α of the PHQ-4 and GAD-2 scale were 0.78 and 0.77 respectively.

Physical health information included the following: physical activity status (“I do not currently exercise and I am not seriously thinking about changing in the next 6 months”, “I exercise sometimes but not regularly” and “I exercise regularly (most days of the week for at least 45 min each time) and have done so for longer than 6 months”); body mass index (BMI); recommended sleeping hours [28]; and pain or discomfort (shoulder, eyes, neck and hands).

### 2.3. Statistical Analysis

Statistical analysis was performed using IBM SPSS Statistics Version 27.0 (Chicago, IL, USA). Univariate statistical analyses were used to determine the prevalence of SA and describe the distribution of sociodemographic characteristics, as well as physical and mental well-being, among participants. A chi-square analysis was conducted to analyse the effect of SA on physical and mental health. In addition, logistic regression analyses were performed to identify the main predictors of SA. Statistical significance was set at a *p* value of <0.05.

## 3. Results

### 3.1. Sociodemographic Characteristics

In total, 440 young adults participated in the study. Over half of the participants were males (53.2%), and 62.7% of the participants were aged ≤25 years old and were students (56.1%). Three quarters of the participants were single (75.0%) and 53.6% of the participants were from a high-income family. The majority of the participants had obtained a college or a university qualification (89.3%) (Table 1).

### 3.2. Smartphone Addiction Prevalence

The overall prevalence of SA among the participants was 61.4%; however, male participants (68.4%) were more addicted compared to female participants (53.4%).

### 3.3. Health Characteristics of the Participants

According to Table 2, 66.6% of the participants reported that they were sometimes physically active as they do exercise sometimes but not regularly. More than one third (34.3%) slept less than 6 h per day. The majority of the participants (81.4%) admitted that their smartphone kept them awake and 63.7% were awake until 12 am because of smartphone use. About 38.4%, 32% and 13% of the participants used their smartphones while eating, walking and driving, respectively. Participants reported experiencing pain in their shoulders (37%), eyes (57.5%), neck (46.1%) and hands (43.6%) as a result of smartphone use.

### 3.4. Associations of Sociodemographics with Smartphone Addiction

#### Univariate Logistic Regression Analysis

Univariate logistic regression analyses were performed to determine the associations between sociodemographics and SA. The analyses found that gender, age, occupation status and family size were significantly associated with SA (Table 3). Male participants were 1.88 times more likely to be addicted to a smartphone compared to their female counterparts (OR = 1.88, 95% CI: 1.28–2.78). Similarly, participants aged ≤25 were 2.88 times more likely to be smartphone-addicted compared to those aged ≥31 (OR = 2.88, 95% CI: 1.50–5.53). In addition, unemployed participants were 2.06 times more likely to be smartphone-addicted in comparison to those who were employed (OR = 2.06, 95% CI: 1.09–3.84). Participants from a large family of ≥8 were 1.42 times more likely to be smartphone-addicted than those living with a small family of ≤4 (OR = 1.42, 95% CI: 1.45–10.72). In conclusion, those who were male, aged ≤25, unemployed, and living with a large family (≥8) were more likely to be smartphone-addicted.

### 3.5. Comparison of Physical Health and Mental Well-Being between Smartphone-Addicted and Non-Addicted Groups

As outlined in Table 4, smartphone-addicted participants were more likely to be physically inactive (26.7%), sleep ≤ 6 h per day (38.1%) and be overweight or obese (32.5%). Participants in the SA group were also more likely to stay awake at night due to smartphone use (88.9%). Participants who were addicted were more likely to use a smartphone while walking (39.3%), driving (31.3%) and eating (42.2%) when compared to the non-addicted group. Smartphone-addicted participants were more likely to feel pain in their shoulders (41.5%), eyes (64.4%), neck (53.0%) and hands (48.1%). Moreover, the chance of having anxiety (40.7%) and depression (40.0%) within the smartphone-addicted group was much higher than in the non-addicted group.

## 4. Discussion

The widespread adaptation of the internet and smartphones has been monumental all around the globe; it has brought profound change to culture and society in both positive and negative ways. Young people have become a more vulnerable group as they spend most of their time on smartphones. This study was an initial step to understanding the association between SA and the sociodemographics and physical and mental well-being of the young adults in Bangladesh. Our findings show that the prevalence of SA among young adults in Bangladesh is 61.4%, which is higher than the global prevalence (26.99%) [29], as well as the prevalence in other countries including Malaysia (46.9%) [8], Turkey (39.8%) [30], Lebanon (44.6%) [31], China (29.8%) [32] and Brazil (33.1%) [33]. Such a high prevalence of SA among the participants is in line with smartphone usage rates in South-East Asia compared to other regions of the world [29]. These rates might be due to the transition from a traditional culture to a “digital culture” in Bangladesh [34], which has seen a sharp increase in smartphone use especially among the younger generation [35]. In addition, young people of the country use smartphones for watching movies or listening to music [36], which could be another possible explanation for the high prevalence of SA among the youth of the country.

In line with the literature, our findings suggest that young adults (aged ≤25) are more likely to be addicted to smartphone use [37,38,39]. One reason for this could be the frequent use of social media via smartphone, which is common among young people [40]. Another probable reason is that young adults who were born and raised during the internet era might prefer online communication to face-to-face communication [41]. In line with previous internet addiction studies in Bangladesh [42,43], this study also revealed that males are more prone to SA. This could be explained by findings that show that males frequently use smartphones for playing online games [44,45]. In contrast to our findings, some studies have reported that females are more prone to SA than males [25,46,47]. This warrants further study to explore what role gender plays in smartphone addiction.

Our study also found that unemployment was associated with SA. Previous research indicates this is likely due to unemployed people having more free time to spend on a smartphone [48]. This current study also revealed that living in a large family (≥8 members) is associated with smartphone overuse, which is similar to the results of a recent study conducted among adolescents in southern China [49]. It is possible that young adults who belong to a large family may have less communication [50] but more conflicts with other family members [49], which may lead to a smartphone overuse. In contrast, other studies have shown that living in a small family is also linked to SA [51,52]. This requires further study to explore how family size impacts smartphone use.

Consistent with previous studies, this study found that SA was more likely to be associated with physical inactivity and being obese or overweight [17,53]. A recent study suggested that smartphone overuse was linked with weight gain [15]. In terms of sleep time, the current study reported that smartphone-addicted participants had less sleep in comparison to non-addicted participants, which is similar to results found in previous studies [30,54,55]. “Bedtime procrastination” [56] and “blue light” [41] emitted by a smartphone are some of the underlying triggering factors of insufficient sleep, as blue light inhibits the brain from releasing melatonin, an important sleep hormone [57,58]. Young adults who are addicted to smartphones may be unable to stop using their smartphones, causing “bedtime procrastination,” which may stimulate poor sleep quality [56]. Like previous studies, the current study also revealed that those who were addicted to smartphones were more likely to use a smartphone while walking and driving [20,59]. This has significant implications for policy makers, as smartphone use while walking and driving can cause serious accidents [20,60].

Our study suggests that smartphone-addicted young adults are more likely to experience physical pain in their shoulders, neck and hands, which is similar to other results in the literature [61,62]. Overuse of smartphones can lead to defective postures that initiate pain in different joints and muscles of the body [63]. In addition, one study reported that “De Quervain tenosynovitis”, or pain in the wrists, was associated with using different electronic devices including smartphones [64]; excessive chatting and texting through a smartphone was among the common risk factors for De Quervain tenosynovitis [65]. Notably, a previous study also revealed that SA can initiate carpal tunnel syndrome [66], which is in line with our findings. This study also reports that SA can lead to eye pain; previous research found that smartphone overuse can induce different eye problems such as redness and blurred vision [67,68], which support our results.

Our findings reveal that young adults those who are addicted to smartphones are more likely to suffer from anxiety and depression when compared to non-addicted groups, which is in line with previous research [7,69,70]. Overusing instant messaging or notifications in social media can lead to more online communication, rather than face-to-face communication, increasing the potential for social isolation [71], which may act as a trigger for anxiety and depression among the younger generations [72,73]. Smartphone-addicted people have a common tendency to check notifications on their mobile phone all the time, which may lead to a “reassurance seeking” pathway [69,74,75], which ultimately results in “Fear of Missing Out (FoMO)”. FOMO has been considered to be one of the main underlying factors of anxiety and depression related to SA [76]. Moreover, numerous studies indicate that SA can lead to nomophobia, which is associated with anxiety and depression [77,78].

The findings from this research are invaluable in identifying the effects of SA, its main drivers and its impact on young adults in Bangladesh. However, this study has some limitations. It was a cross-sectional study; therefore, a causal relationship cannot be determined. The data were self-reported, which is a potential source of bias. In addition, data were collected through convenience sampling methods, so the findings may not be generalizable for the Bangladesh-wide population of young adults.

## 5. Conclusions

This study found a high prevalence of SA among young adults in Bangladesh, which has significant implications for policy makers with respect to the health outcomes of young adults both at present and into the future. Our finding suggest that there is a need to find solutions to address the significant impact of SA at a national and international level. National and international public health organizations should develop policies and guidelines to reduce smartphone overuse. Furthermore, it is important to raise awareness of the potential long-term effects of SA on the health outcomes of young adults.

## Figures and Tables

**Table 1:** Distribution of sociodemographic characteristics, N = 440.

	n	(%)
Gender		
Male	234	(53.2)
Female	206	(46.8)
Age ^a^		
≤25	276	(62.7)
26–30	120	(27.3)
≥31	44	(10.0)
Occupation status ^b^		
Employed	127	(20.9)
Unemployed	66	(15.0)
Students	247	(56.1)
Family monthly income		
<5000 TK	39	(8.9)
5000–<10,000 TK	56	(12.7)
10,000–<20,000 TK	109	(24.8)
≥20,000 TK	236	(53.6)
Marital status ^b^		
Single	330	(75.0)
Married	103	(23.4)
Others (Divorced and Widowed)	7	(1.6)
Family size ^a^		
Small (≤4)	204	(46.4)
Average (5–7)	206	(46.8)
Large (≥8)	30	(6.8)
Education level ^b^		
Primary or secondary	47	(10.7)
College or university qualification	393	(89.3)

^a^ “Age and family size were continuous variables but they are categorized into groups for the purpose of this study”. ^b^ “Occupation status, marital status and education level categories are re categorized due to small numbers in some original categories”.

**Table 2:** Distribution of health characteristics, N = 440.

	n	(%)
Physical activity		
I do not currently exercise	98	(22.3)
I exercise sometimes	293	(66.6)
I exercise regularly	49	(11.1)
Average of sleep hours ^a^		
≤6 h (Not Recommended)	151	(34.3)
7–9 h (Recommended)	270	(61.4)
≥10 (Not Recommended)	19	(4.3)
Is your smartphone keep you awake at night		
Yes	358	(81.4)
No	82	(18.6)
If yes, up to		
12 am	130	(36.3)
After 12 am	228	(63.7)
Do you use your smartphone while walking?		
Yes	141	(32.0)
No	299	(68.0)
Do you use your smartphone while driving?		
Yes	57	(13.0)
No	193	(43.2)
Not applicable	190	(43.2)
Do you eat while using smartphone?		
Yes	169	(38.4)
No	271	(61.6)
Body Mass Index (BMI) ^b^		
Underweight (≤18.4)	47	(10.7)
Healthy weight (18.5–24.9)	271	(61.6)
Overweight (25.0–29.9)	89	(20.2)
Obese (≥30.0)	33	(7.5)
Experienced pain		
Shoulder		
Yes	163	(37.0)
No	277	(63.0)
Eyes		
Yes	253	(57.5)
No	187	(42.5)
Neck		
Yes	203	(46.1)
No	237	(53.9)
Hands		
Yes	192	(43.6)
No	248	(56.4)
PHQ-4 ^c^		
Anxiety (GAD-2)		
Anxiety	155	(35.2)
Non-Anxiety	285	(64.8)
Depression (PHQ-2)		
Depressed	147	(33.4)
Non-Depressed	293	(66.6)

^a^ Average sleep time was a continuous variable but it is categorized into “recommended” and “not recommended” times according to the Sleep Foundation (https://www.sleepfoundation.org/press-release/national-sleep-foundation-recommends-new-sleep-times, accessed on 1 June 2022). ^b^ BMI was calculated by using participants’ weight and height data. ^c^ GAD-2 and PHQ-2 Scores of ≥3 indicate a positive screening for anxiety and depression symptoms.

**Table 3:** Univariate logistic regression analysis of associations of sociodemographic variables with smartphone addiction, N = 440.

	Non-Smartphone Addicted		Smartphone Addicted		OR	95% CI		*p*
	n	(%)	n	(%)		Lower	Upper	
Gender								
Male	74	(43.5)	160	(59.3)	1.88	1.28	2.78	0.001
Female	96	(56.5)	110	(40.7)	Ref.			
Age								
≤25	92	(54.1)	184	(68.1)	2.88	1.50	5.53	0.001
26–30	52	(30.6)	68	(25.2)	1.88	0.93	3.80	0.075
≥31	26	(15.3)	18	(6.7)	Ref.			
Occupation status								
Employed	60	(35.3)	67	(24.8)	Ref.			
Unemployed	20	(11.8)	46	(17.0)	2.06	1.09	3.84	0.025
Students	90	(52.9)	157	(58.1)	1.56	1.01	2.41	0.044
Family monthly income								
<5000 TK	14	(8.2)	25	(9.3)	Ref.			
5000–<10,000 TK	21	(12.4)	35	(13.0)	0.93	0.39	2.18	0.873
10,000–<20,000 TK	39	(22.9)	70	(25.9)	1.00	0.46	2.15	0.990
≥20,000 TK	96	(56.5)	140	(51.9)	0.81	0.40	1.65	0.573
Marital status								
Single	120	(70.6)	210	(77.8)	Ref.			
Married	48	(28.2)	51	(20.4)	0.65	0.41	1.02	0.064
Divorced/Widowed/Others	2	(1.2)	5	(1.9)	1.42	0.27	7.47	0.673
Family size								
Small (≤4)	90	(52.9)	114	(42.2)	Ref.			
Average (5–7)	75	(44.1)	131	(48.5)	1.37	0.92	2.04	0.112
Large (≥8)	5	(2.9)	25	(9.3)	1.42	1.45	10.72	0.007
Education level								
Primary or secondary	19	(11.2)	28	(10.4)	Ref.			
College or university qualification	151	(88.8)	242	(89.6)	1.08	0.58	2.01	0.790

OR = odds ratio, CI = confidence interval, A *p*-Value ≤ 0.05 was considered significant.

**Table 4:** Comparison between physical health and mental well-being information between smartphone-addicted and non-smartphone-addicted groups, N = 440.

	Non-Smartphone Addicted		Smartphone Addicted		df	X^2^ (440)	*p*
**Physical Activity**	**n**	**(%)**	**n**	**(%)**	2	9.711	0.008
I do not currently exercise	26	(15.3)	72	(26.7)			
I exercise sometimes	119	(70.0)	174	(64.4)			
I exercise regularly	25	(14.7)	24	(8.9)			
Average of sleep hours ^a^					2	6.657	0.036
≤6 h (Not Recommended)	48	(28.2)	103	(38.1)			
7–9 h (Recommended)	111	(65.3)	159	(58.9)			
≥10 (Not Recommended)	11	(6.5)	8	(3.0)			
Is your smartphone keep you awake at night?					1	26.09	0.001
Yes	118	(69.4)	240	(88.9)			
No	52	(30.6)	30	(11.1)			
If yes, up to					1	5.632	0.018
12 am	53	(44.9)	77	(32.1)			
After 12 am	65	(55.1)	163	(67.9)			
Do you use your smartphone while walking?					1	16.70	0.001
Yes	35	(20.6)	106	(39.3)			
No	135	(79.4)	164	(60.7)			
Do you use your smartphone while driving?					1	14.62	0.001
Yes	11	(10.7)	46	(31.3)			
No	92	(89.3)	101	(68.7)			
Do you eat while using smartphone?					1	4.295	0.038
Yes	55	(32.4)	114	(42.2)			
No	115	(67.6)	156	(57.8)			
Body Mass Index (BMI) ^b^					3	9.204	0.027
Underweight (≤18.4)	22	(12.9)	25	(9.3)			
Healthy weight (18.5–24.9)	114	(67.1)	157	(58.1)			
Overweight (25.0–29.9)	23	(13.5)	66	(24.4)			
Obese (≥30.0)	11	(6.5)	22	(8.1)			
Experienced pain							
Shoulder					1	5.896	0.015
Yes	51	(30.0)	112	(41.5)			
No	119	(70.0)	158	(58.5)			
Eyes					1	13.79	0.001
Yes	79	(46.5)	174	(64.4)			
No	91	(53.5)	96	(35.6)			
Neck					1	13.10	0.001
Yes	60	(35.3)	143	(53.0)			
No	110	(64.7)	127	(47.0)			
Hands					1	5.784	0.016
Yes	62	(36.5)	130	(48.1)			
No	108	(63.5)	140	(51.9)			
PHQ-4							
Anxiety					1	9.310	0.002
Anxiety	45	(26.5)	110	(40.7)			
Non-Anxiety	125	(73.5)	160	(59.3)			
Depression					1	13.64	0.001
Depressed	39	(22.9)	108	(40.0)			
Non-Depressed	131	(77.1)	162	(60.0)			

^a^ Average of sleep hours was a continuous variable but it is categorized into recommended and not recommended times according to Sleep Foundation (https://www.sleepfoundation.org/press-release/national-sleep-foundation-recommends-new-sleep-times, accessed on 1 June 2022). ^b^ BMI was calculated by using participation’s weight and height data.

## Data Availability

The data presented in this study are available on request from the corresponding author. The data are not publicly available due to privacy restrictions.
